# Developing automated methods for disease subtyping in UK Biobank: an exemplar study on stroke

**DOI:** 10.1186/s12911-021-01556-0

**Published:** 2021-06-15

**Authors:** Kristiina Rannikmäe, Honghan Wu, Steven Tominey, William Whiteley, Naomi Allen, Cathie Sudlow, the UK Biobank

**Affiliations:** 1 Centre for Medical Informatics, University of Edinburgh, NINE Edinburgh BioQuarter, 9 Little France Road, Edinburgh, EH16 4UX UK; 2 Health Data Research UK, London, UK; 3 Institute of Health Informatics, University College London, London, UK; 4 Medical School, University of Edinburgh, Edinburgh, UK; 5 Centre for Clinical Brain Sciences, University of Edinburgh, Edinburgh, UK; 6 Nuffield Department of Population Health, University of Oxford, Oxford, UK; 7 UK Biobank, Stockport, UK; 8 BHF Data Science Centre, London, UK

**Keywords:** Natural language processing, Disease subtyping, Stroke, Cerebral hemorrhage, Brain scan

## Abstract

**Background:**

Better phenotyping of routinely collected coded data would be useful for research and health improvement. For example, the precision of coded data for hemorrhagic stroke (intracerebral hemorrhage [ICH] and subarachnoid hemorrhage [SAH]) may be as poor as < 50%. This work aimed to investigate the feasibility and added value of automated methods applied to clinical radiology reports to improve stroke subtyping.

**Methods:**

From a sub-population of 17,249 Scottish UK Biobank participants, we ascertained those with an incident stroke code in hospital, death record or primary care administrative data by September 2015, and ≥ 1 clinical brain scan report. We used a combination of natural language processing and clinical knowledge inference on brain scan reports to assign a stroke subtype (ischemic vs ICH vs SAH) for each participant and assessed performance by precision and recall at entity and patient levels.

**Results:**

Of 225 participants with an incident stroke code, 207 had a relevant brain scan report and were included in this study. Entity level precision and recall ranged from 78 to 100%. Automated methods showed precision and recall at patient level that were very good for ICH (both 89%), good for SAH (both 82%), but, as expected, lower for ischemic stroke (73%, and 64%, respectively), suggesting coded data remains the preferred method for identifying the latter stroke subtype.

**Conclusions:**

Our automated method applied to radiology reports provides a feasible, scalable and accurate solution to improve disease subtyping when used in conjunction with administrative coded health data. Future research should validate these findings in a different population setting.

**Supplementary Information:**

The online version contains supplementary material available at 10.1186/s12911-021-01556-0.

## Background

UK Biobank (UKB) is a prospective population-based cohort study with extensive phenotypic and genotypic information on > 500,000 participants (www.ukbiobank.ac.uk). It is an open access resource, established to facilitate research into the determinants of a wide range of health outcomes [1]. Disease outcomes are ascertained primarily via linkages to routinely collected coded national administrative health datasets [2], enabling the identification of a broad range of disease phenotypes with sufficient accuracy for many research studies [2–4]. However, these coded data are often incomplete and less accurate when it comes to identifying specific disease subtypes [2, 3]. For example, up to 40% of participants with a stroke code in hospital, death record or primary care administrative data in UKB do not have a code specifying their stroke subtype, even though review of the full text medical records shows that a stroke subtype was known in over 99% of cases [2]. Further, among subtype specific codes, hemorrhagic stroke codes may have precision as low as 42% [2]. This will be a limitation for many researchers since stroke is a heterogeneous disease, and genetic and environmental risk factors to date have been found to be very subtype specific. Indeed, the International Stroke Genetics Consortium has already identified stroke subtyping as a top research priority [3]. Similarly, while coded data can be used to identify all-cause dementia, accuracy in identifying dementia subtypes, in particular vascular dementia, is much lower [4, 5]. This may be a limitation for researchers studying genetic and environmental associations specific to disease subtypes, and hence automated, scalable methods are urgently needed to improve disease subtyping.

Possible solutions to enhance the accuracy of coded data and improve the ability to deep-phenotype (e.g. subtype) all participants at scale include linkage to national disease-specific audit and registry datasets and/or the development of automated tools to extract data from participants’ detailed electronic medical records (EMR). While linkage to disease-specific datasets is promising, these data are limited to select diseases, may not cover all regions or nations of the UK, may cover limited time periods, do not always capture primary care and outpatient as well as inpatient encounters, and may have unknown accuracy. On the other hand, approaches relying on mining the complete EMR are limited by the challenges of data anonymization and of accessing the many different systems used by hospitals and other regional healthcare providers across the UK. For diseases that are diagnosed based on imaging, an alternative approach would be to access not the complete EMR but only the participants’ relevant clinical radiology reports. Since imaging reports are stored in more accessible and unified data repositories (including, in the UK, national Picture Archiving and Communications Systems in Scotland [6] and Wales [7] and seven regional imaging networks in England [8]) and contain far less text than the entire EMR, both research access and anonymization of these data are likely to be much less challenging.

Inferring disease subtypes from free text is challenging for computers, as it is usually beyond the scope of named entity recognition tasks. For example, inferring that the combination of the two entities “bleeding” and “intracerebral” signifies intracerebral hemorrhage (ICH) requires clinical knowledge. While deep learning methods have great potential to learn such associations, large datasets would be required to train them. At the same time, many disease subtypes are rare by nature, which is a limitation for supervised learning.

By combining natural language processing (NLP) with clinical knowledge inference, this work aimed to investigate the feasibility and added value of automated methods applied to clinical radiology reports in ascertaining accurate disease subtype information for participants with any stroke code in a regional UKB subpopulation. We used stroke as an exemplar disease, specifically looking to improve hemorrhagic stroke identification. Stroke patients always require brain imaging to exclude alternative diagnoses and determine the stroke subtype, although ischemic stroke is not always visible on imaging done very soon after symptom onset [9].

## Methods

### Study population

**Fig. 1:**
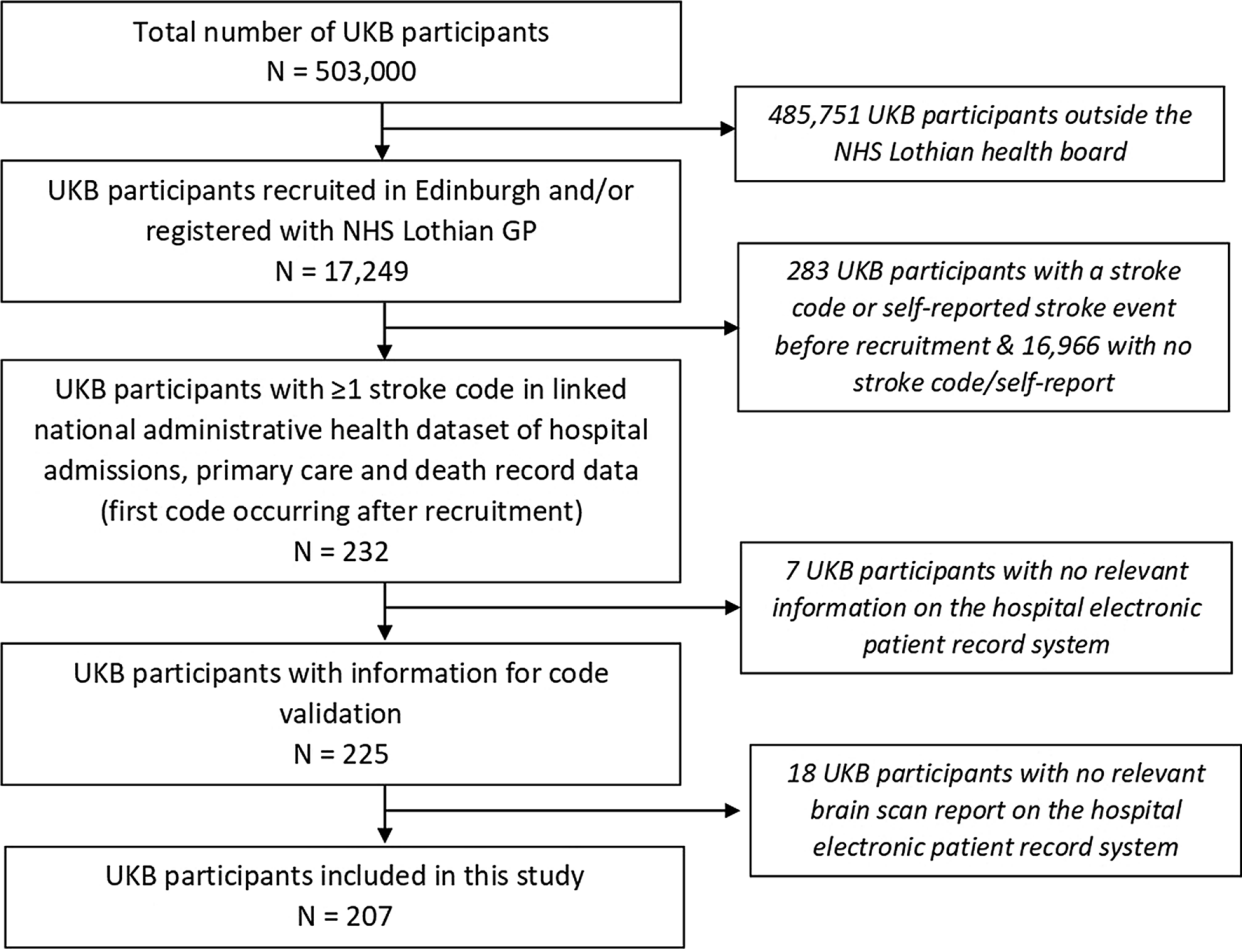
Selection of included UK Biobank (UKB) participants. *GP* general practitioner, *NHS* National Health Service; Information for code validation refers to the participant having any information on the hospital electronic patient record system to allow an expert stroke physician to confirm or reject the accuracy of the coded diagnosis [2]

We conducted the study in a sub-population of 17,249 UKB participants in the Lothian region of southeast Scotland. All participants’ records were linked to national administrative health datasets, providing hospital, death record and primary care administrative coded data. Within this UKB sub-population, we identified participants with ≥ 1 stroke code in their linked health data, indicating a stroke diagnosis after their recruitment to UKB. ICD-10 and Read v2 codes that were used to identify stroke cases are available in Additional file 1: Table S1. The follow-up period was from the participant’s date of recruitment up to the end of September 2015, the date at which data were complete for all sources at the time of this study. Clinicians screened the participants’ EMR and extracted all clinical brain scan reports (MRI, CT, CTA, MRA, DSA) available relating to the respective codes (Fig. 1). Further detail about the study population is published elsewhere [2].

### Automated methods applied to clinical brain scan reports to derive stroke subtypes

**Fig. 2:**
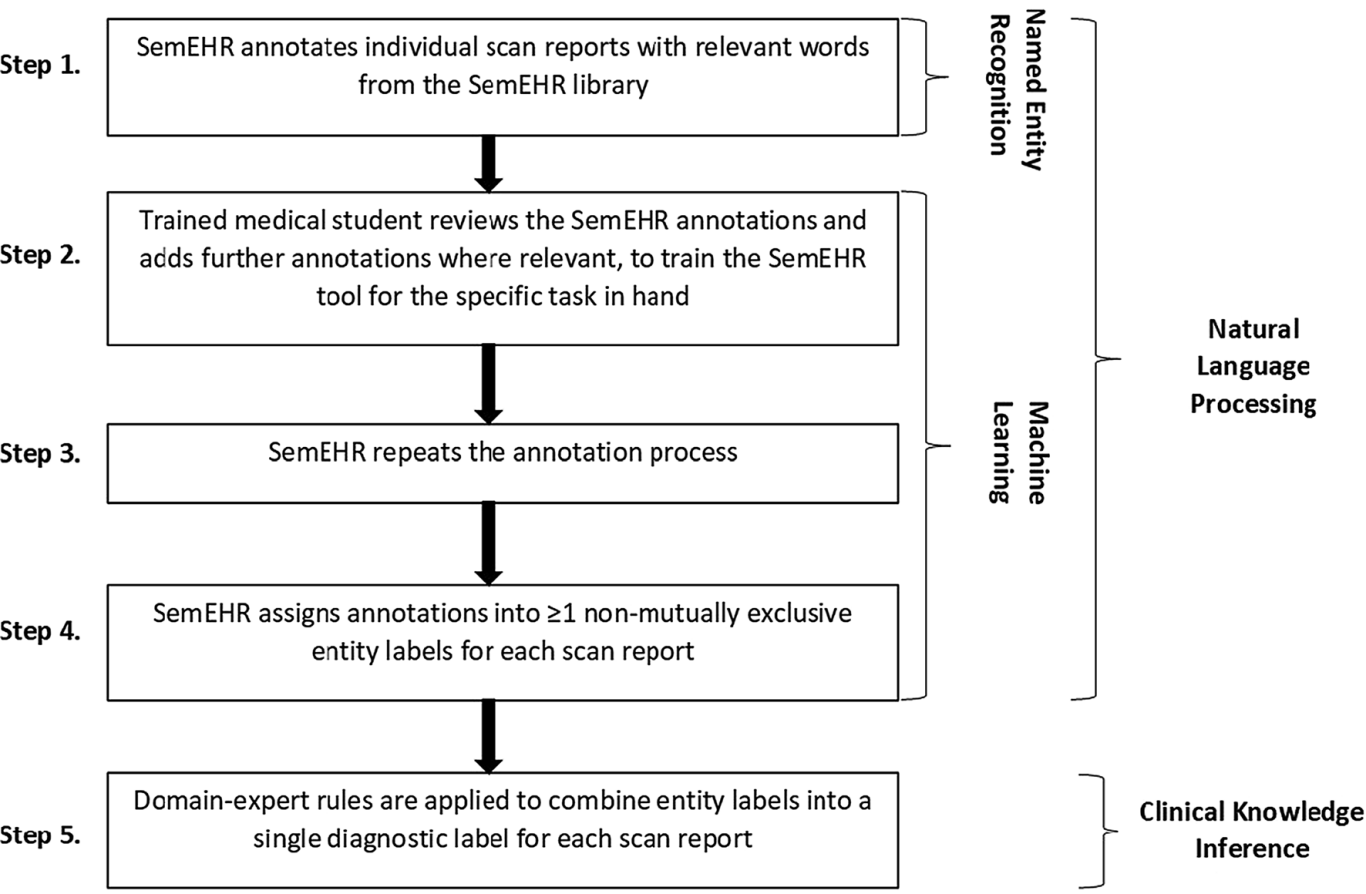
Pipeline for automated disease subtyping based on clinical scan reports. The medical student who undertook the scan report annotations to train the Sem-EHR tool for the current task was a final year medical student who had completed their clinical neurology and stroke modules. They spent time reading the literature around the topic and practiced scan report annotation under the training of a neurologist before the study

The automated method pipeline was composed of five steps (detailed in Fig. 2), including NLP (named entity recognition followed by machine learning) followed by applying clinical knowledge inference. We used an off-the-shelf tool—SemEHR [10], developed and trained on EMR in UK National Health Service (NHS) Trusts in London, and further extended on Scottish imaging datasets [11]. We derived entity labels for each scan report (full list is available in the first column of Table 3 in the “Results” section).

Steps 1–4 in Fig. 2 illustrate the process of identifying relevant entity labels for each scan report. Essentially, it comprised two phases. First, it used a baseline NLP model from SemEHR to obtain an initial set of annotation results, which were contextualized (positive/negated/hypothetical/history mention/not a phenotype) mentions of Unified Medical Language System [12] concepts (detailed in [10]). More detail about contextualizing with examples is provided in Additional file 1: Table S2. A mapping was then used to retain relevant mentions (those were mapped to the list in Table 3) and only positive mentions were retained. In the second phase, two authors (neurologist KR and medical student ST), blinded to each other’s decisions, annotated a randomly selected subset of 40 reports to check agreement. Inter-annotator agreement was substantial (90% agreement, k 0.735) and all scan reports were then annotated by one author (medical student ST) (step 2 in Fig. 2). We used these human annotations to further improve the NLP model via SemEHR’s continuous learning framework, adopting a tenfold cross validation for the further improvement and validation (results reported in Table 3).

Once entity labels were assigned to each scan, we then used clinical knowledge inference on the entity labels to infer a single diagnostic stroke subtype label for each scan report: primary intracerebral hemorrhage [ICH], primary subarachnoid hemorrhage [SAH], primary ischemic stroke [IS] (step 5 of Fig. 2, full list of rules in Table 1). If a participant had more than one scan report, and the inferred diagnostic stroke subtype labels were different across these reports, the participant was classified into all inferred stroke subtype categories for the subsequent analyses. Such rules encode clinical knowledge to infer participant-level stroke subtypes in a computable format and are directly reusable in new settings. Although we considered the more common reasons for a false-positive stroke/stroke subtype diagnosis in our specific dataset when developing the domain-expert rules, the underlying principles are not unique to this dataset and hence we expect them to be generalizable across other stroke datasets. The methods and rules are publicly available [13].

**Table 1:** Domain-expert rules to combine entity labels into a single diagnostic label for each scan report

Diagnostic labels	Inclusion reasons	Exclusion reasons
ICH	Presence of entity label: (a) intracerebral haemorrhage	Presence of ≥ 1 entity labels: (a) metastatic tumour or tumour; (b) contusion; (c) time recent and ischaemic stroke; (d) transformation; (e) subarachnoid haemorrhage + aneurysm (f) subdural haematoma
SAH	Presence of entity label: (a) subarachnoid haemorrhage	Presence of ≥ 1 entity labels: (a) metastatic tumour or tumour; (b) contusion; (c) transformation; (d) intracerebral haemorrhage if no mention of aneurysm; (e) subdural haematoma
IS	Presence of entity labels: (a) time recent and (b) ischaemic stroke	

British English spelling was used for entity labels in the original study. ICH, primary intracerebral hemorrhage; SAH, primary subarachnoid hemorrhage, IS, primary ischemic stroke

### Data analyses

We calculated the proportion of participants with a stroke code where automated methods could assign a stroke subtype based on clinical brain scan report(s).

#### Precision and recall of automated methods in assigning a stroke subtype among all UKB participants with any stroke code

For automated stroke subtype diagnoses at a participant level, we calculated precision (or positive predictive value, i.e. the percentage of participants allocated a particular stroke subtype by the automated method who were true positives for that subtype) and recall (or sensitivity, i.e. the percentage of participants who truly had a particular stroke subtype detected by the automated method). We used stroke physician adjudications derived based on the participants’ complete EMR (including, but not limited to the clinical brain scan reports) as the ground-truth. We calculated 95% confidence intervals (CIs) using the exact Clopper-Pearson method in StatsDirect [14]. We compared the results of the automated method to the results of using administrative disease codes alone based on our earlier work [2]. We also calculated the entity label-level precision and recall values, using physician annotated scan reports as the ground truth.

#### Precision and recall of automated methods in assigning a stroke subtype among UKB participants with a hemorrhagic stroke subtype code

We next limited our analyses to participants with a hemorrhagic stroke subtype code. Our previous work found that the hemorrhagic stroke subtype code recalls were 100% but precision was much lower, ranging from 42% for ICH to 71% for SAH [2]. We hypothesized that automated methods applied to specifically to participants with a hemorrhagic stroke code would improve the accuracy of the coded data without sacrificing the recall.

#### Predicting the best possible performance of automated methods in assigning a stroke subtype among UKB participants with a hemorrhagic stroke subtype code

To understand the best results that an automated approach could potentially achieve, we further investigated if clinical brain scan reports contain the necessary information for a human expert to assign a hemorrhagic stroke subtype. To study this, we performed a further round of expert stroke physician adjudications, asking an expert to assign a stroke subtype based on the clinical brain scan report. This was done by 2 expert adjudicators blinded to each other’s adjudications to assess inter-adjudicator agreement. We compared the results to the ground truth obtained from stroke expert physician adjudications based on the complete EMR (including, but not limited to the clinical brain scan reports).

## Results

Of the 225 cases with a stroke code in our UKB sub-population, 207 (92%) had a relevant clinical brain scan report available (Fig. 1). The number of cases in each diagnostic category as annotated by experts is provided in Additional file 1: Table S3a. Of these 207 cases, 72 had multiple relevant reports, and the total number of unique reports analyzed was 352. Only one of the 72 participants with multiple relevant reports was assigned to two different stroke subtype categories (ICH and SAH) with the automated methods.

### Data analyses

Overall, scan report-based automated methods were able to assign a stroke subtype to 149 of the 207 cases (72%) (Additional file 1: Table S3b). 113 of these 149 assigned subtypes were confirmed by experts as being accurate (PPV 76%). This means that automated methods can assign an accurate stroke subtype in 55% (113/207) of cases.

#### Precision and recall of automated methods in assigning a stroke subtype among all UKB participants with any stroke code

Participant-level results of automated methods showed very good precision for ICH (89%; 8 true-positive among 9 identified cases), good precision for SAH (82%; 14 true-positive among 17 identified cases) and moderate precision for IS (73%; 91 true-positive among 124 identified cases) diagnoses, representing a significant improvement in precision compared with coded data for ICH (89% vs 42%), a slight improvement for SAH (82% vs 71%) and a worsening for IS (73% vs 83%). Recall was very good for ICH (89%), good for SAH (82%) and moderate for IS (64%), representing a slight worsening for ICH and SAH (100% vs 82% and 89%), and a slight improvement for IS (49% vs 64%) compared with coded data. (Table 2).

**Table 2:** Participant-level diagnostic label precision and recall estimates against reference standard (i.e. expert physician adjudications based on the complete EMR)

Stroke subtype	Precision (i.e. positive predictive value) (95% CI)		Recall (i.e. sensitivity) (95% CI)	
	From codes (based on previous work [2])	From automated method	From codes (based on previous work [2])	From automated method
ICH	42% (31–54%) (11/26)	89% (52–100%) (8/9)	100% (72–100%) (11/11)	89% (52–100%) (8/9)
SAH	71% (54–83%) (17/24)	82% (57–96%) (14/17)	100% (80–100%) (17/17)	82% (57–96%) (14/17)
IS	83% (75–89%) (73/88)	73% (65–81%) (91/124)	49% (41–57%) (73/149)	64% (56–72%) (91/142)
IS (including cases with an unspecified subtype assigned as IS)	80% (76–83%) (147/184)	77% (71–83%) (141/182)	99% (95–100%) (147/149)	99% (96–100%) (141/142)

ICH, intracerebral hemorrhage; SAH, subarachnoid hemorrhage; IS, ischemic stroke; IS (including cases with an unspecified subtype assigned as IS) = all cases where a stroke subtype could not be assigned with automated methods or where the code was unspecified for a stroke subtype were assumed to be ischemic stroke; Precision = positive predictive value (proportion of true-positive cases among all cases). Recall = sensitivity (proportion of all true-positive cases in the population identified). Absolute numbers of cases provided in brackets. The dataset used for the precision and recall calculation from codes in our previous work [2] included a total of 225 participants with a stroke code. The dataset used for the precision and recall calculation from automated method in this study includes a total of 207 participants with a stroke code. The 207 are a subset of the 225 participants with a stroke code who also had a relevant clinical brain scan report available. 18 participants among the 225 participants did not have a brain scan available and were hence excluded from this study

Entity-level precision and recall estimates were good, ranging from 78 to 100%, and from 83 to 100%, respectively (Table 3).

**Table 3:** Entity label-level precision and recall estimates

Concept mentions	Precision (%)	Recall (%)
Metastatic tumor	93	87
Aneurysm	97	100
Intracerebral haemorrhage	95	100
Time old (temporal words/phrases indicating old events, e.g., *old* ischemic stroke)	78	83
Subdural haematoma	97	83
Contusion	100	100
Subarachnoid haemorrhage	90	100
Related to (words/phrases indicating relations between two events, e.g., bleeding *because of* a recent fall)	78	100
Ischaemic stroke	90	99
Time recent (temporal words/phrases indicating recent events, e.g., *accute* ischemic stroke)	91	97
Meningioma	100	100
Transformation	88	100
Traumatic	100	75

Numbers are mean values of tenfold cross validation

#### Precision and recall of automated methods in assigning a stroke subtype among UKB participants with a hemorrhagic stroke subtype code

When limiting the analyses to participants with a hemorrhagic stroke code, precision improved for SAH from 82 to 88%, but remained unchanged for ICH. As expected, the recall estimates were unchanged (Table 4).

**Table 4:** Participant-level diagnostic label precision and recall estimates among those with a hemorrhagic stroke code

	Precision (95% CI)	Recall (95% CI)
ICH	89% (52–100%) (8/9)	89% (52–100%) (8/9)
SAH	88% (62–98%) (14/16)	82% (57–96%) (14/17)

ICH, intracerebral hemorrhage; SAH, subarachnoid hemorrhage; Precision = positive predictive value (proportion of true-positive cases among all cases). Recall = sensitivity (proportion of true-positive cases identified among all true-positive cases). Absolute numbers of cases provided in brackets

#### Predicting the best possible performance of automated methods in assigning a stroke subtype among UKB participants with a hemorrhagic stroke subtype code

Expert adjudication of scan reports showed only slightly improved results compared to automated methods applied on scan reports, with precision of 90% and 100%, and recall of 100% and 88% for ICH and SAH respectively (Additional file 1: Table S4). This suggests that (a) the information contained in the clinical brain scan reports is sufficient to assign a hemorrhagic stroke subtype in the majority of participants and (b) automated methods are only slightly inferior to the human adjudicator. Only the first expert adjudicator’s results are reported here since inter-adjudicator agreement for assigning a stroke subtype was very good at 97%.

## Discussion

Our results demonstrate the potential for significant added value and feasibility of using automated methods on clinical brain scan reports to improve stroke subtyping in UKB. While the automated method assigned a correct stroke subtype diagnosis to only 55% cases overall, its main benefit came from markedly improving the precision of hemorrhagic stroke codes. As expected, ischemic stroke code accuracy remained similar. This approach of combining NLP and clinical knowledge inference is potentially scalable across the UK and may also scale well in other settings. It may also be relevant to disease subtyping for other conditions, where information from images is important in the diagnosis of disease subtypes. Furthermore, the SemEHR tool used in this project can be easily adapted for research into other phenotypes by adopting transfer learning technologies [15].

Compared to coded data alone, for hemorrhagic stroke, the automated method improved precision at the expense of slightly poorer recall. Depending on the study design, more importance can be attributed to either estimate, however our achieved trade-off is likely to be preferrable for many research studies. For ischemic stroke, the effect was the opposite, resulting in a lower precision at the expense of improved recall. An additional caveat is that the true-positive ischemic stroke cases identified by the automated method are likely to be different to the true-positive cases missed. This is because cases identified will have had a stroke resulting in a visible lesion on the scan, and hence are likely to be clinically more severely affected. Therefore, for ischemic stroke, unless the automated method can achieve a near-perfect recall, many research studies are likely to prefer using coded data to avoid this bias.

As a substantial proportion of clinical features are only available in free text [16], NLP has been extensively studied and applied to extract clinical features from medical records [17–29]. Methodologies used range from rule-based approaches [23, 27] to machine learning approaches [17, 22, 24–26] to deep learning methods [18]. However, to date most of the work has focused on named entity recognition tasks, such as semantics in domain terminologies (e.g. ontology-driven inferences) [10, 28] and identification of contextual mentions (e.g. negation, temporality and the person to whom the information refers to) [15, 18]. Very few studies [17] have investigated methods to help derive disease sub-phenotypes from free text, where the information to derive these exists but additional clinical knowledge is needed to derive it. Our work addressed this gap by combining NLP with clinical knowledge inference. Advantages of this approach are that it does not require the very large datasets required to train machine learning methods, along with the potential both to transfer knowledge to new datasets from external sources and to apply the approach in other languages. This is currently a relatively understudied area, with very few sharable resources available.

Previous studies applying NLP and machine learning to classify stroke into subtypes have focused on automating ischemic stroke subtyping into specific sub-categories using the EMR [30, 31] or a selection of available features [32]. Others, such as the Edinburgh Information Extraction for Radiology reports (EdIE-R) [33] have shown good performance of text mining systems in subtyping already expert-validated stroke cases into the three main subtypes (IS, ICH and SAH) based on radiology scan reports. Our study differs from these in two main ways. Firstly, it is nested in a population-based cohort study rather than a disease specific cohort. We combine existing information from national administrative health datasets with automated methods by identifying participants with a high prior probability of having had a true-positive stroke diagnosis (represented by them having a stroke code in the administrative data) followed by the application of automated methods to subtype stroke into the three main types (IS, ICH and SAH). This approach means that the results are applicable to other population-based studies and large biobanks using administrative data for disease identification (e.g. the UK-based Generation Scotland [34] study and SAIL Dataset [35]). Secondly, we use expert stroke physician adjudications based on the complete EMR to derive ground truth diagnoses. This step is important, since while in a large number of cases the correct hemorrhagic stroke subtype diagnosis can be reached by the expert based only on the brain scan report, in a proportion of cases, additional information from the complete EMR is required in addition. One example of this would be a case where a patient’s brain scan report describes a brain hemorrhage, which could be secondary to head injury (i.e. a traumatic hemorrhage, not a stroke) or it could be a primary hemorrhage (i.e. a stroke), and additional medical history regarding any mention of a relevant traumatic event prior to symptom onset in the EMR will help make the correct final diagnosis. We are not aware of any previous studies combining these two features in order to automate stroke subtyping.

Our results show that in large population-based cohorts, the ascertainment of cases via codes indicating stroke combined with subsequent automated methods applied to the free text of brain scan reports is a feasible and potentially scalable approach for enhancing the accuracy of stroke subtyping. Our primary approach was to first identify participants with a high prior probability of having had a stroke of any subtype (defined as participants with any stroke code in administrative datasets) and then apply automated methods to enhance the accuracy of specific subtype diagnosis (IS vs ICH vs SAH). We also explored the benefit of identifying participants with a high prior probability of having had a hemorrhagic stroke subtype (defined as participants with a hemorrhagic stroke specific code in administrative datasets) before applying automated methods. This improved the precision of SAH subtyping slightly, but would need to be validated in larger datasets.

The strengths of our study include the application and testing of existing methods on a real-world dataset. In addition, we tested the performance of the methods against robust ground-truth diagnoses made by specialist physicians based on the complete EMR. To maximize the reusability of our work, we deliberately decoupled the NLP component from the clinical knowledge inference component in our pipeline, so that the latter can be reused in different settings. We have also made the model and inference rules publicly available [13] to facilitate future similar studies by others. The imaging reports however are currently only available for UKB sub-cohorts via individual data linkage projects.

Our study also has some limitations. The relatively wide confidence intervals for precision and recall suggest a high variability of these estimates, which could be due to the small sample size and heterogenicity of the sample, particularly for the SAH and ICH cases. Also, this work didn’t have a replication or an external validation cohort for evaluating the pipeline. Furthermore, in our study, we included only participants’ first-ever stroke events and their relevant clinical brain scans were selected manually by experts, whereas this step would also need to be automated to make the approach scalable in large datasets. We envisage this may involve including all brain scans within a certain timeframe from the stroke code. Finally, we did not apply a rule-based approach to tackle the issue of some participants having multiple brain scans with competing disease subtypes. Developing methods to address this may improve the performance of automated methods further.

Further work to build on these results is now needed and should focus on: (1) validating our automated methods in further datasets, which could include additional UK Biobank sub-cohorts as well as data from other population-based cohorts; (2) investigating the time interval between the code(s) and clinical scan reports to enable inclusion of the most relevant data; (3) investigating the usefulness of the automated methods in identifying recurrent stroke events; (4) developing rules for disease subtype adjudication based on multiple reports per participant; and (5) expanding this work to investigate disease subtyping of other conditions beyond stroke.

## Conclusions

We have developed an automated pipeline which can be applied to clinical scan reports to enable significantly improved stroke subtyping. Furthermore, we demonstrate the feasibility and scalability of this approach, as well as its potential future application to a much wider range of phenotypes.

## Supplementary Information

**Additional file 1.** Supplementary material.

## Data Availability

We have made the model and inference rules publicly available at https://github.com/CogStack/nlp2phenome to facilitate future similar studies by others. The imaging reports used in this study however are currently only available for specific UKB approved data linkage projects. Please contact the corresponding author for any queries.

## Abbreviations

ICH: Intracerebral hemorrhage
SAH: Subarachnoid hemorrhage
IS: Ischemic stroke
UKB: UK Biobank
EMR: Electronic medical records
NLP: Natural language processing
NHS: National Health Service
CT: Computed tomography
MRI: Magnetic resonance imaging
CTA: CT angiography
MRA: MR angiography
DSA: Digital subtraction angiography
CI: Confidence interval
EdIE-R: Edinburgh Information Extraction for Radiology reports
GP: General physician
