# Supplementary material for: Developing automated methods for disease subtyping in UK Biobank: an exemplar study on stroke
Source: BMC Med Inform Decis Mak. 2021 Jun 15;21:191. doi: 10.1186/s12911-021-01556-0 (PMC8204419; doi:10.1186/s12911-021-01556-0)
Supplement: Supplementary file 1 — Additional file 1. Supplementary material. [file 12911_2021_1556_MOESM1_ESM.docx]

**SUPPLEMENTARY MATERIAL**

**Supplementary Table 1**. ICD10 and Read v2 stroke codes

**Supplementary Table 2**. Contextualized phenotype mentions

**Supplementary Table 3.**

a. Number of cases in each diagnostic category as annotated by experts

b. Number of cases in each diagnostic category as annotated by automated method

**Supplementary Table 4**. Human expert assigning a stroke subtype based on the clinical brain scan report alone

**Supplementary Table S1**. ICD10 and Read v2 stroke codes

| **ICD10 text** | **ICD10 code** |
| --- | --- |
| **Stroke type specific codes** | |
| Subarachnoid haemorrhage | I60 |
| Subarachnoid haemorrhage from carotid siphon and bifurcation | I60.0 |
| Subarachnoid haemorrhage from middle cerebral artery | I60.1 |
| Subarachnoid haemorrhage from anterior communicating artery | I60.2 |
| Subarachnoid haemorrhage from posterior communicating artery | I60.3 |
| Subarachnoid haemorrhage from basilar artery | I60.4 |
| Subarachnoid haemorrhage from vertebral artery | I60.5 |
| Subarachnoid haemorrhage from other intracranial arteries | I60.6 |
| Subarachnoid haemorrhage from intracranial artery, unspecified | I60.7 |
| Other subarachnoid haemorrhage | I60.8 |
| Subarachnoid haemorrhage, unspecified | I60.9 |
| Intracerebral haemorrhage | I61 |
| Intracerebral haemorrhage in hemisphere, subcortical | I61.0 |
| Intracerebral haemorrhage in hemisphere, cortical | I61.1 |
| Intracerebral haemorrhage in hemisphere, unspecified | I61.2 |
| Intracerebral haemorrhage in brain stem | I61.3 |
| Intracerebral haemorrhage in cerebellum | I61.4 |
| Intracerebral haemorrhage, intraventricular | I61.5 |
| Intracerebral haemorrhage, multiple localized | I61.6 |
| Other intracerebral haemorrhage | I61.8 |
| Intracerebral haemorrhage, unspecified | I61.9 |
| Cerebral infarction | I63 |
| Cerebral infarction due to thrombosis of precerebral arteries | I63.0 |
| Cerebral infarction due to embolism of precerebral arteries | I63.1 |
| Cerebral infarction due to unspecified occlusion or stenosis of precerebral arteries | I63.2 |
| Cerebral infarction due to thrombosis of cerebral arteries | I63.3 |
| Cerebral infarction due to embolism of cerebral arteries | I63.4 |
| Cerebral infarction due to unspecified occlusion or stenosis of cerebral arteries | I63.5 |
| Cerebral infarction due to cerebral venous thrombosis, nonpyogenic | I63.6 |
| Other cerebral infarction | I63.8 |
| Cerebral infarction, unspecified | I63.9 |
| **Unspecified stroke codes** | |
| Stroke, not specified as haemorrhage or infarction | I64 |
| Stroke, not specified as haemorrhage or infarction, accompanying other code | I64X |
| **Read v2 text** | **Read v2 code** |
| **Stroke type specific codes** | |
| Subarachnoid haemorrhage | G60.. |
| Ruptured berry aneurysm | G600. |
| Subarachnoid haemorrhage from carotid siphon and bifurcation | G601. |
| Subarachnoid haemorrhage from middle cerebral artery | G602. |
| Subarachnoid haemorrhage from anterior communicating artery | G603. |
| Subarachnoid haemorrhage from posterior communicating artery | G604. |
| Subarachnoid haemorrhage from basilar artery | G605. |
| Subarachnoid haemorrhage from vertebral artery | G606. |
| Subarachnoid haemorrhage from intracranial artery, unspecified | G60X. |
| Subarachnoid haemorrhage NOS. | G60z. |
| Subarachnoid haemorrhage from other intracranial arteries | Gyu60 |
| Subarachnoid haemorrhage from intracranial artery, unspecified | Gyu6E |
| Other subarachnoid haemorrhage | Gyu61 |
| Intracerebral haemorrhage | G61.. |
| Cortical haemorrhage | G610. |
| Internal capsule haemorrhage | G611. |
| Basal nucleus haemorrhage | G612. |
| Cerebellar haemorrhage | G613. |
| Pontine haemorrhage | G614. |
| Bulbar haemorrhage | G615. |
| External capsule haemorrhage | G616. |
| Intracerebral haemorrhage, intraventricular | G617. |
| Intracerebral haemorrhage, multiple localised | G618. |
| Lobar cerebral haemorrhage | G619. |
| Intracerebral haemorrhage in hemisphere, unspecified | G61X. |
| Left sided intracerebral haemorrhage, unspecified | G61X0 |
| Right sided intracerebral haemorrhage, unspecified | G61X1 |
| Intracerebral haemorrhage NOS. | G61z. |
| Other intracerebral haemorrhage | Gyu62 |
| Intracerebral haemorrhage in hemisphere, unspecified | Gyu6F |
| Cerebral infarction due to thrombosis of precerebral arteries | G63y0 |
| Cerebral infarction due to embolism of precerebral arteries | G63y1 |
| Cerebral arterial occlusion | G64.. |
| Cerebral embolism | G641. |
| Cerebral thrombosis | G640. |
| Cerebral infarction due to thrombosis of cerebral arteries | G6400 |
| Cerebral infarction due to embolism of cerebral arteries | G6410 |
| Cerebral infarction NOS | G64z. |
| Brainstem infarction | G64z0 |
| Wallenberg syndrome | G64z1 |
| Left sided cerebral infarction | G64z2 |
| Right sided cerebral infarction | G64z3 |
| Infarction of basal ganglia | G64z4 |
| Cerebral infarction due to unspecified occl/stenosis of precerebral arteries | G6W.. |
| Cerebral infarction due to unspecified occl/stenosis of precerebral arteries | Gyu6G |
| Cerebral infarction due to unspecified occl/stenosis of cerebral arteries | G6X.. |
| Cerebral infarction due to unspecified occl/stenosis of cerebral arteries | Gyu63 |
| Cerebral infarction due to cerebral venous thrombosis, non pyogenic | G6760 |
| Other cerebral infarction | Gyu64 |
| **Unspecified stroke codes** | |
| Stroke and cerebrovascular accident unspecified | G66.. |
| Middle cerebral artery syndrome | G660. |
| Anterior cerebral artery syndrome | G661. |
| Posterior cerebral artery syndrome | G662. |
| Brainstem stroke syndrome | G663. |
| Cerebellar stroke syndrome | G664. |
| Pure motor lacunar syndrome | G665. |
| Pure sensory lacunar syndrome | G666. |
| Left sided CVA | G667. |
| Right sided CVA | G668. |
| Cerebral palsy, not congenital or infantile, acute | G669. |

**Supplementary Table 2**. Contextualized phenotype mentions.

This study uses SemEHR to identify mentions of phenotypes from scan reports and to classify the context of each mention into 5 categories.

| **Examples** | **Types of phenotype mentions** | |
| --- | --- | --- |
| visible acute stroke | Contextualised  mentions | positive mention |
| with no evidence of stroke recurrence |  | negated mention |
| is concerning for brain tumour |  | hypothetical mention |
| a sign of old bleeding |  | history mention |
| patient asked for information about stroke | not a phenotype mention | |

**Supplementary Table 3.**

a. Number of cases in each diagnostic category as annotated by experts

| **Expert diagnosis** | **Number of cases in each category as annotated by experts** |
| --- | --- |
| ICH | 9 |
| SAH | 17 |
| IS | 142 |
| False-positive stroke code | 39 |
| **Total number of participants included** | **207** |

b. Number of cases in each diagnostic category as annotated by automated method

| **Automated method diagnosis** | **Number of cases in each category as annotated by automated method** |
| --- | --- |
| ICH | 9* |
| SAH | 17* |
| IS | 124 |
| No subtype assigned | 58 |
| **Total number of participants included** | **207** |

*One participant was assigned to both ICH and SAH diagnostic category.

ICH = intracerebral hemorrhage; SAH = subarachnoid hemorrhage; IS = ischemic stroke

**Supplementary Table 4**. Human expert* assigning a stroke subtype based on the clinical brain scan report alone

| **Stroke subtype** | **Precision (95% CI)** | **Recall (95% CI)** |
| --- | --- | --- |
| ICH | 90% (56% to 100%)  (9/10) | 100% (66% to 100%)  (9/9) |
| SAH | 100% (78 to 100%)  (15/15) | 88% (64% to 99%)  (15/17) |

*Inter-rater agreement for stroke subtype among cases of stroke was 97%. ICH = intracerebral hemorrhage; SAH = subarachnoid hemorrhage; CI = confidence interval
